# Reframing Critical Needs in Vector Biology and Management of Vector-Borne Disease

**DOI:** 10.1371/journal.pntd.0000566

**Published:** 2010-02-23

**Authors:** Shirley Luckhart, Steven W. Lindsay, Anthony A. James, Thomas W. Scott

**Affiliations:** 1 Department of Medical Microbiology and Immunology, University of California at Davis, Davis, California, United States of America; 2 School of Biological and Biomedical Sciences, Durham University, Durham, United Kingdom; 3 Departments of Microbiology and Molecular Genetics, Molecular Biology and Biochemistry, University of California at Irvine, Irvine, California, United States of America; 4 Department of Entomology, University of California at Davis, Davis, California, United States of America; Fogarty International NIH, United States of America

## Background

Recent advances in empirical, methodological, and theoretical aspects of vector biology are an impetus for reexamining critical research needs aimed at improving human health. The discipline of vector biology is characterized by its emphasis on disease prevention, and successes are well documented. Vector interventions were essential for reduction of malaria and yellow fever in the 1950s and 1960s, dengue in Singapore and Cuba [1], and onchocerciasis in West Africa [2]. Unfortunately, victories are too often the exception, or when they do occur they are difficult to sustain. Malaria remains among the biggest infectious disease killers; lymphatic filariasis has proven difficult to eliminate; Chagas disease, African trypanosomiasis, onchocerciasis, and leishmaniasis are underserved; dengue continues to expand its geographic range; and West Nile and chikungunya viruses invaded new continents with little resistance [3],[4]. Although vector control remains an essential component in the battle against vector-borne disease (VBD), persistence of vector-borne pathogens and resilience of their arthropod vectors continue to motivate the search for novel solutions.

In the past two decades vector biologists have responded to this challenge by reassessing the status of research in their field [5]–[8]. Contributions range from an Institute of Medicine report that, 17 years ago, identified VBDs as among the most important emerging microbial threats to the United States [9] to the current call for fundamental shifts in areas of emphasis, execution, and application of VBD research [10]. Reviews revealed progress in some areas (e.g., genomics, genetics, and quantitative analyses), while accomplishments in other areas lagged behind expectations (e.g., field evaluation of transgenic vectors and development of insecticides with novel modes of action). Common to all of these reports is the premise that the overall goal should be to reduce or prevent pathogen transmission and disease. Adjustments in sequential reports dealt with the perceived best path to reaching that objective. Across these reviews, recommendations can be distilled to five major needs: (1) novel intervention tools (e.g., new public health insecticides, biological control agents, and genetics-based instruments [11],[12]); (2) improved disease prevention strategies (e.g., integrating different vector control strategies and combining vector control with other prevention tools, such as drugs and vaccines, to attack multiple VBDs [1],[13]); (3) enhanced surveillance methods and data analysis; (4) broader integration of scientific subdisciplines (e.g., vector biology, clinical research, natural and social environmental biology); and (5) expanded training opportunities [12],[14].

## Identifying Central Issues in VBD Control

Despite these well-intended recommendations, VBD prevention continues to be challenged by incomplete coordination among individuals with complementary expertise, inability to implement long-term solutions, and reluctance to embrace the complexity of vector biology and pathogen transmission systems in intervention strategies. In this context, an international meeting of vector biologists (participants are listed in the acknowledgments section) was convened at the University of California, Davis, in January 2008 to develop a revised agenda based on the principal theme of improving integration in VBD management. The meeting format emphasized: (1) redefining common challenges and opportunities across a range of VBDs; (2) initiating and sustaining strategically planned interactions among investigators within and among a diversity of research areas; and (3) developing a working list of research areas that merit increased attention.

## A Challenge Issued

Consistent with the “working list” concept we present the following challenge to our readers: the content of this article must be modified through vigorous and open discussion of supportive, dissenting, and divergent opinions to consolidate ownership of a collective agenda that results in action, reaction, collaboration, and change. We appreciate the support of the Public Library of Science to moderate these first steps through submission of online comments and discussion by members of our community and others who would like to contribute to this process.

## Themes and Strategies

The most significant outcome of the meeting was the identification of three overarching themes: integration, sustainability, and heterogeneity. *Integration* of tools and strategies is necessary to increase the potential for improved public health outcomes across different diseases, transmission ecologies, and epidemiologic settings. In these contexts, *sustainability* of VBD prevention is essential. Although some short-term victories are necessary, greater emphasis should be placed on programs anticipated to have a sustained positive public health impact. *Heterogeneity* is a fundamental component of patterns and processes in VBD transmission. Consequently, variation in the biology, ecology, and genetics of vectors and pathogens and the impact of these phenomena on pathogen transmission and disease must be more fully accounted for in integrated programs to ensure continued, successful disease prevention.

By combining the strengths of different approaches, a specific strategy can act faster, last longer, and be leveraged against more than one vector or disease. The theory and practice of integrated pest management purport that multidimensional strategies are superior to a single, narrowly focused approach [15]. This is consistent with the World Health Organization (WHO) expanded concept for integrated vector management (IVM) for vector control using currently available technology, but also endorses new and continued research on pathogen transmission dynamics and tools and strategies for disease prevention that will fundamentally and significantly improve public health. IVM is defined as “a rational decision-making process for the optimal use of resources for vector control” [16] and is based on the use of sustainable vector control methods alone or in combination to reduce contact between humans and vector arthropods [16]. Sustainability derives from cost-effective decision-making that enfranchises participants at the community level and that is integrated at the national and international levels [17],[18]. Where reasonable, IVM approaches should be integrated with clinical interventions, epidemiological studies, public health management of water availability and quality, and management of land-use and agricultural pests, which collectively influence VBD transmission, to achieve control that is economically, socially, and ecologically sustainable.

The discussion that follows is intended to be a cross-sectional view of VBD research in contrast to more traditional “siloed” approaches that are defined by individual diseases or vector species. Details of research areas identified as requiring additional attention are provided as Supporting Information (Tables S1, S2, S3, S4). Selected research examples are used to illustrate key points of the three overarching themes.

## Integration

### Control Methods and Strategies

Integrated control methods that target multiple vectors and multiple diseases and focus disease prevention on interrupting human-vector contact are urgently needed (Table S2, C). An example is *La Casa Segura* or “the safe home” concept [19], an intervention based on delivery of insecticides into domestic dwellings, which are assumed to be the primary site of human–insect interaction and thus pathogen transmission. This approach and methods like it have not been evaluated in large-scale efforts against a specific disease (e.g., malaria or dengue), while simultaneously leveraging reductions in a variety of NTDs (e.g., Chagas disease, human African trypanosomiasis, and leishmaniasis) and insect pest species.

Integration of entomological, epidemiological, and ecological (both natural and social) data in risk models is needed to optimize location- and situation-specific control strategies (Table S1, B; Table S3, C). The Pacific Program to Eliminate Lymphatic Filariasis is an example of an intervention that evolved into an integrated program. Initial efforts were based on mass drug administration, but now include vector control. The integrated approach is showing greater impact than when only drugs were used and after the programmatic vision was expanded to include lymphatic filariasis, malaria, and dengue. Similarly, the Southern Cone initiative to eliminate Chagas disease from endemic South America is a case study in successful integration [20]. Public health professionals and vector biologists integrated surveillance of blood supplies and donor screening with triatomine vector control and surveillance, transmission modeling, and community-based educational programs for sustainable Chagas disease control at the community level across an array of ecological settings [20]. Evolution of Chagas control towards ecosystem management [21] is a concept that merits evaluation for other VBDs.

Ideally, field-collected data are integrated in real time into decision support systems – which include mathematical, simulation, or statistical models (Table S3, C) – that can inform appropriate control strategies for specific conditions and geographical areas [19]. In this way vector control programs can adapt strategies to the unique features of local conditions, and systematically predict the benefits of integration across interventions (e.g., drugs, vaccines, and efforts to manage coinfections that alter host immunity [22] Table S1, A; Table S4, B). Surveillance tools and analytical models, within decision support systems, should help to identify interventions that might inadvertently enhance pathogen transmission [23]; they should also be sufficiently flexible to accommodate emerging or resurging pathogens and vectors (Table S2, B; Table S4, E). Emerging VBDs can arise through changes in pathogen virulence, the introduction of new vectors or vertebrate hosts, or anthropogenic changes [24] and, therefore, can be difficult to identify. For example, during the initial stages of its invasion into North America, West Nile virus was misidentified as St. Louis encephalitis virus [25].

Newer approaches and technologies (e.g., novel insecticides and repellents and genetic strategies) will by necessity be transitioned into existing programs that are composed of extant tools (e.g., biocontrol agents, surveillance tools and methods, and modeling) [26]. A key missing technology for vector control is rapid, high-throughput detection of insecticide resistance (Table S2, A). Such detection is needed because the frequency of resistant alleles in a population as measured by current technology can increase rapidly from undetectable to very common, such that resistance cannot be reversed. Therefore, management of insecticide resistance should be a priority to extend the useful life of currently available and future insecticides [11].

There is a significant need for integration across control programs to assess different strategies and tools in different public health, epidemiologic, and ecologic settings (Table S2, C). Although some aspects of control will require approaches that are case-specific, cooperation and communication across a broad spectrum of control programs allows the identification of “common denominators” that have not been harnessed for maximum benefit. In an analogous fashion, there is an opportunity to join the movement toward integrated interventions that are based on the geographic overlap of high-prevalence NTDs. For example, NTD clinical management can be enhanced by integrating disease-specific drug administration efforts and creating a “rapid impact” package of free or low-cost generic drugs [27]. In particular, filariasis, onchocerciasis, schistosomiasis, soil-transmitted helminth infections, and trachoma could be managed in sub-Saharan Africa by concurrent provision of four drugs. This measure would save time and money and provide maximum health benefit to populations that are difficult to access [28]. As practitioners, vector biologists should study these approaches to determine when, where, and how they could improve existing VBD prevention programs.

### Research

A greater emphasis on applied research and the application of basic research in disease endemic settings would be an important step in closing the gap between field and laboratory research (Table S4, D). Although efforts have been made to integrate work from laboratory to field and from field to laboratory, more progress is possible and needed (Table S1, A). It is important to validate laboratory-based results in the field (Table S4, E), where vectors naturally transmit pathogens. On the other hand, laboratory studies often can be more elegantly controlled than field studies and when informed by field data can be used to develop useful model systems. Although this concept has been a goal for some time, it has seldom been realized. Adding to the difficulty in addressing this challenge is the fact that there are few if any templates for how to achieve this kind of integration, particularly in academic settings where advanced training has become increasingly compartmentalized and narrowly focused [12]. One notable success in this regard is the Multilateral Initiative on Malaria (MIM), which was established in 1997 as a component of the United Nations Children's Fund (UNICEF) - United Nations Development Programme (UNDP) - World Bank - World Health Organization Special Programme for Research and Training in Tropical Diseases (WHO TDR). The stated mission of MIM is “to strengthen and sustain through collaborative research and training, the capacity of malaria-endemic countries in Africa to carry out research that is required to develop and improve tools for malaria control and to strengthen the research-control interface.” The MIM has also embraced bureaucratic and regulatory challenges, which must be accounted for in any VBD management plan because they continue to be significant barriers that discourage reciprocal exchanges between laboratory and field [29] (Table S3, B). MIM has increased the priority of malaria on political agendas to secure additional resources for education and control.

The effectiveness and sustainability of control interventions will require greater recognition and incorporation of the integrated nature of vector physiology into control programs (Table S4, B). For example, there is significant metabolic “cross-talk” among mechanisms underlying insecticide resistance, lifespan, reproduction, and immunity, and among the regulatory pathways that mediate these processes [30]–[32]. Strategies that target a single physiology will likely influence others. Consequently, this interplay among physiological processes demands attention in research on the biology and control of vectors (Table S4, C).

### Scientific Meetings and Training

Although vector biologists can choose from a variety of scientific meetings to attend, few emphasize higher-level interactions across disciplines. VBD researchers would benefit from disease-specific scientific meetings that require researchers to communicate across epidemiology, systems biology, modeling, vector biology and control, health system delivery, and social sciences and to network with researchers engaged in research on water management, agriculture, vaccine and drug development, and diagnostics. The goal should be to encourage scientists to step outside of their comfort zones so that they develop new lines of communication that are critical for holistic management of hosts, pathogens, and the environment. Collaboration across disciplines will substantially improve the prospects for sustainable VBD control.

Training programs that are focused exclusively on vectors and their biology and ecology are few and available to a relatively small group of students. For 18 years the Biology of Disease Vectors (BDV) course (http://www.cvmbs.colostate.edu/mip/bdv/), supported by UNICEF-UNDP-World Bank-WHO TDR and the John D. and Catherine T. MacArthur Foundation, served as an example of the effectiveness of broad-based vector biology training [8]. The course provided intensive training in vector biology/ecology for young scientists from around the world that inspired new ideas and collaborations that lasted long after the course had ended. The BDV course is an example of the positive impact that training programs can have when they are adequately supported, both financially and intellectually, by the research community. Because the course is no longer available in its original form there is an urgent need for new international training programs to stimulate and engage young vector biologists (Table S3, A). Such programs should integrate disciplines and provide broad training for a large number of investigators, with the goal of improving networking and collaboration among scientists from disease-endemic and nonendemic countries.

An underutilized and too often overlooked resource for bridging integration is the combined strengths of academic and government/military research units (Table S3, B). Collaboration across these units can enhance and extend academic training and also stabilize and enhance government/military research and overseas laboratories. Government and military overseas laboratories with traditional strengths in VBD research have resources and well-established field sites that are unique, often in disease-endemic areas. Recently, some of these programs have seen significant reductions in funding and shifts in focus away from VBDs; they may be in danger of elimination [12]. Conversely, academic VBD training has struggled to provide the invaluable hands-on experience that exists at government and military overseas laboratories. Harnessing the combined strengths of academic, government, and military laboratories will result in an enriched environment for students and trainees.

## Sustainability

### Infrastructure, Resources, and Advocacy

Sustainability of successful VBD interventions and the research that supports those efforts will depend on strong intellectual and physical infrastructure, stakeholder enfranchisement, and political buy-in. In disease-endemic countries, the ability to sustain effective VBD prevention requires local expertise; e.g., continued training at all levels, development of professional degree programs (thesis and nonthesis MSc as well as PhD), and professionalization of vector control as a discipline (Table S3, A). Infrastructure and resources could be leveraged across much of the VBD-endemic world by developing and maintaining stock and strain storage centers for pathogens and vectors, providing greater access to vector infection assays and containment facilities, offering on-line courses and resources, opening access to well-characterized field sites, and continuing expansion of publicly available technology and reference reagent centers in endemic and nonendemic countries (Table S3, B). The National Institutes of Health National Center for Research Resources, which serves many research areas, could function as a prototype for the development of additional VBD-specific research resources. Success of these kinds of programs will require scientific and policy advocacy groups/programs that effectively promote interventions and required funding to decision-makers, policy-makers, and the people who implement those programs. Research can contribute by providing advocates with data and concepts that are needed to effectively argue for enhanced VBD research.

## Heterogeneity

### Host, Pathogen, and Environment

Heterogeneity is increasingly recognized as having fundamentally powerful impacts on infectious disease dynamics [32],[33]. Compared to non-vector-borne pathogens, the insertion of vector into the transmission cycle exponentially increases the complexity of the system. Heterogeneities derive from the natural and social environment (spatial and temporal), from the organisms in the VBD cycle, and from coinfecting pathogens. Rapid expansion of human populations and anthropogenic change drive the need for expanded capacities to monitor, analyze, and model heterogeneity.

As with most difficult problems, there is a desire to identify simple solutions. However, the growing disease burden, despite decades of effort, is a testament to pathogen and vector persistence and to the resilience of VBD cycles. Enhanced computational power constitutes an opportunity for vector biologists to more fully embrace heterogeneity in the organisms and systems they study and to more effectively use that knowledge to improve VBD surveillance and prevention programs (Table S1, A/B). Ultimately, these advances can be implemented as decision support systems that are sufficiently user friendly and flexible to be available in real time and to reflect changing social, biological, economic, ethical, and medical needs. For example, natural heterogeneity in mosquito movement [33], human movement [34], preferred hosts, and the individuals who contribute most to infection [35],[36] can be used to map spatial and temporal patterns of mosquito-borne diseases and, hence, to improve the efficacy of control efforts.

## Conclusions

In light of current global changes in VBD transmission and in technological and research advancements, a reevaluation of the VBD research agenda is needed. This must be a community-based evolution that results in effective and sustainable disease control, not the production of a static document that rapidly becomes obsolete and irrelevant. The vision of the future is broader and more holistic than the past, looking at new, multiple, and integrated methods for combating VBDs. Success will require that vector biologists more effectively engage with clinicians, epidemiologists, and other natural and social scientists. The future requires breaking down silos, thinking about using combinations of tools for disease control, and attacking multiple diseases. Although some portions of this agenda have been reviewed previously, we have focused on those issues that remain relevant, added new ones, and have attempted to move past those that are no longer relevant. Indeed, we seek to stimulate a new discussion and new actions for the discipline of vector biology.

## Supporting Information

Table S1 Ecological, biological, and societal aspects of transmission.
(0.10 MB DOC)

Table S2 Tools and interventions.
(0.10 MB DOC)

Table S3 Resources and models.
(0.10 MB DOC)

Table S4 Basic science of relevance to vector-borne diseases.
(0.11 MB DOC)

## References

[1] Morrison AC, Zielinski-Gutierrez E, Scott TW, Rosenberg R (2008). Defining challenges and proposing solutions for control of the virus vector *Aedes aegypti*.. PLoS Med.

[2] Resh VH, Lévêque C, Statzner B (2004). Long-term, large-scale biomonitoring of the unknown: assessing the effects of insecticides to control river blindness (onchocerciasis) in West Africa.. Annu Rev Entomol.

[3] WHO/TDR (2007). 18^th^ Programme Report: Tropical disease research progress 2005–2006.

[4] Gould EA, Higgs S (2009). Impact of climate change and other factors on emerging arbovirus diseases.. Trans R Soc Trop Med Hyg.

[5] WHO/TDR (2002). Insect Vectors and Human Health. Report of the Scientific Working Group meeting held 12–16 August 2002.

[6] Smolinski MS, Hamburg MA, Lederberg J (2003). Microbial Threats to Human Health: Emergence, Detection and Response.

[7] Institute of Medicine, Board on Global Health (2008). Vector-Borne Diseases: Understanding the Environmental, Human Health, and Ecological Connections - Workshop Summary.

[8] Beaty BJ, Prager DJ, James AA, Jacobs-Lorena M, Miller LH (2009). From Tucson to genomics and transgenics: The vector biology network and the emergence of modern vector biology.. PLoS Negl Trop Dis.

[9] Lederberg J, Shope RE, Oaks SC, editors (1992). Emerging Infections: Microbial Threats to Health in the United States.

[10] Lambrechts L, Knox TB, Wong J, Liebman KA, Albright RG (2009). Shifting priorities in vector biology to improve control of vector-borne disease.. Trop Med Int Health.

[11] Hemingway J, Beaty BJ, Rowland M, Scott TW, Sharp BL (2006). The Innovative Vector Control Consortium: improved control of mosquito-borne diseases.. Trends Parasitol.

[12] Morens DM (2008). Confronting vector-borne diseases in an age of ecologic change.. IOM Workshop Summary. Vector-Borne Diseases: Understanding the Environmental, Human Health, and Ecological Connections.

[13] WHO (2007). Report of the WHO consultation on integrated vector management (IVM)..

[14] Hotez PJ (2004). Should we establish a North American school of global health sciences?. Am J Med Sci.

[15] Shea K, Thrall PH, Burdon JJ (2000). An integrated approach to management in epidemiology and pest control.. Ecol Lett.

[16] WHO (2004). Global strategic framework for integrated vector management.

[17] van den Berg H, Takken W (2007). A framework for decision-making in integrated vector management to prevent disease.. Trop Med Int Health.

[18] van den Berg H, Takken W (2009). Evaluation of integrated vector management.. Trends Parasitol.

[19] Eisen L, Beaty BJ (2008). Innovative decision support and vector control approaches to control dengue.. IOM. Vector-Borne Diseases – Understanding the Environmental, Human Health, & Ecological Connections.

[20] Dias JC (2007). Southern Cone Initiative for the elimination of domestic populations of *Triatoma infestans* and the interruption of transfusional Chagas' disease. Historical aspects, present situation, and perspectives.. Mem Inst Oswaldo Cruz.

[21] Boischio A, Sánchez A, Orosz Z, Charron D (2009). Health and sustainable development: challenges and opportunities of ecosystem approaches in the prevention and control of dengue and Chagas' disease.. Cad Saude Publica.

[22] Gopinath R, Ostrowski M, Justement SJ, Fauci AS, Nutman TB (2000). Filarial infections increase susceptibility to human immunodeficiency virus infection in peripheral blood mononuclear cells in vitro.. J Infect Dis.

[23] Lloyd-Smith JO, Poss M, Grenfell BT (2008). HIV-1/parasite co-infection and the emergence of new parasite strains.. Parasitology.

[24] Molyneux DH (2003). Common themes in changing vector-borne disease scenarios.. Trans R Soc Trop Med Hyg.

[25] Asnis DS, Conetta R, Teixeira AA, Waldman G, Sampson BA (2000). The West Nile virus outbreak of 1999 in New York: the Flushing Hospital experience.. Clin Infect Dis.

[26] Marshall JM, Taylor CE (2009). Malaria control with transgenic mosquitoes.. PLoS Med.

[27] Hotez PJ (2009). Mass drug administration and integrated control for the world's high-prevalence neglected tropical diseases.. Clin Pharmacol Ther.

[28] Hotez PJ, Molyneux DH, Fenwick A, Kumaresan J, Sachs SE (2007). Control of neglected tropical diseases.. N Engl J Med.

[29] Knols BGJ, Louis C (2006). Bridging laboratory and field research for genetic control of disease vectors..

[30] McCarroll L, Paton MG, Karunaratne SH, Jayasuryia HT, Kalpage KS (2000). Insecticides and mosquito-borne disease.. Nature.

[31] Luckhart S, Riehle MA (2007). The insulin signaling cascade from nematodes to mammals: insights into innate immunity of *Anopheles* mosquitoes to malaria parasite infection.. Dev Comp Immunol.

[32] Lazzaro BP, Little TJ (2009). Immunity in a variable world.. Philos Trans R Soc Lond B Biol Sci.

[33] Smith DL, Dushoff J, McKenzie FE (2004). The risk of a mosquito-borne infection in a heterogeneous environment.. PLoS Biol.

[34] Stoddard ST, Morrison AC, Vasquez-Prokopec GM, Kitron U, Paz-Soldan V (2009). The role of human movement in the transmission of vector-borne pathogens.. PLoS Negl Trop Dis.

[35] Lloyd-Smith JO, Schreiber SJ, Kopp PE, Getz WM (2005). Superspreading and the effect of individual variation on disease emergence.. Nature.

[36] Woolhouse ME, Dye C, Etard JF, Smith T, Charlwood JD (1997). Heterogeneities in the transmission of infectious agents: implications for the design of control programs.. Proc Natl Acad Sci U S A.

