# Supplementary material for: Reframing Critical Needs in Vector Biology and Management of Vector-Borne Disease
Source: PLoS Negl Trop Dis. 2010 Feb 23;4(2):e566. doi: 10.1371/journal.pntd.0000566 (PMC2826393; doi:10.1371/journal.pntd.0000566)
Supplement: Table S1 — Ecological, biological, and societal aspects of transmission. (0.10 MB DOC) [file pntd.0000566.s001.doc]

| **A. Vectors^1^** |
| --- |
| 1. Increase studies utilizing the natural range of genetic heterogeneity of vector populations and vector-pathogen interactions to identify potential impacts on vector-borne disease agent transmission. |
| 1. Adapt studies of vectors and pathogens beyond the single vector-single pathogen model to better understand the effects of co-infections on pathogen transmissibility, vector competence, and disease. |
| 1. Iteratively incorporate knowledge from field studies to improve laboratory-based disease models for controlled studies of mechanisms, pathways, and impacts of transmission-blocking interventions. |
| **B. Environmental/Societal Factors and Transmission Dynamics** |
| 1. Assess the impact of environmental change (e.g., agriculture, deforestation, water management, climate change) on vector population dynamics, heterogeneity, and pathogen transmission. |
| 1. Increase quality and quantity of large datasets from long-term longitudinal studies to map seasonal, temporal, and spatial distributions and abundances of pathogens, vectors and reservoirs and to forecast dynamics in transmission and epidemics. |
| 1. Develop systems for geo-spatial data management and distribution to improve identification and mapping of determinants of disease risk and outbreaks. |
| 1. Improve inclusion of social scientists and economists in the evaluation and implementation of control strategies and in communications with stakeholders and policy makers. |

^1^Tables S1-S4 summarize research and training needs identified by participants of the January 2008 Workshop on Emerging Barriers to the Management of Vector-borne Diseases. This material is best viewed as a “work-in-progress” rather than a definitive inventory of research priorities.
