# Supplementary material for: Reframing Critical Needs in Vector Biology and Management of Vector-Borne Disease
Source: PLoS Negl Trop Dis. 2010 Feb 23;4(2):e566. doi: 10.1371/journal.pntd.0000566 (PMC2826393; doi:10.1371/journal.pntd.0000566)
Supplement: Table S2 — Tools and interventions. (0.10 MB DOC) [file pntd.0000566.s002.doc]

| **A. Insecticides and Repellents** |
| --- |
| 1. Optimize insecticide-based control under different transmission conditions (climate, vector species, landscape, housing, ecology, epidemiology, etc) with emphasis on the use of improved formulations and application procedures. |
| 1. Develop high-throughput assays for identification of novel control agents that might be exploited by industry to bring new products to the market. Emphasis should be placed on slow-acting insecticides that reduce development of resistance, on biological control agents and new repellents as alternatives to pesticides, and on emerging technology of biocides or bio-degradable surfactants for vector control. |
| 1. Identify the mechanisms of action of old and new insecticides and repellents to enhance screening for resistance and to predict effectiveness of novel combinations to extend the life of available materials. |
| 1. Identify metabolic pathways and protein targets that might be worthy candidates for insecticide/biocide development. |
| 1. Develop improved sampling protocols, assays and models for monitoring and predicting spread of insecticide resistance with a goal of detecting the earliest indicators of resistance. |
| 1. Determine the impact of emergence of insecticide resistance on disease prevention and identify the operational level of resistance that indicates failure of a control intervention. |
| 1. Develop decision support systems – with complementary interfaces that are usable by scientists, policy makers and public health officials – to assess different application strategies, control strategy failure, and whether insecticide resistance contributed to control failure. |
| **B. Diagnostics, Assays and Surveillance Tools** |
| 1. Develop more sensitive surveillance tools for vector populations and pathogens to monitor and evaluate control strategies, to identify new and emerging diseases, to predict pathogen dynamics and disease outbreaks, and to determine the relative spatial and temporal impacts of interventions. |
| 1. Develop improved assays for precise discrimination of vector species (e.g., species in multi-member complexes, sibling species). |
| **C. Implementation of Control and Intervention Strategies** |
| 1. Support and enhance Integrated Vector Management (IVM). That is, develop new approaches for control of vector-borne diseases through the integration of available control tools and allied interventions (e.g., clinical, educational) into broader management programs. |
| 1. Investigate the additive/synergistic effects of multiple control strategies to enhance IVM and reduce resistance to insecticides or other control agents. |
| 1. Improve the translation of basic research into control strategies and the translation of novel control strategies into operationally effective methods. |
| 1. Enhance public education on disease prevention strategies through iterative analysis of perceptions of success and adverse events that can impact implementation and sustainability of control programs. |
| 1. Develop broad scale programs for vectors/agents that are currently lacking (e.g., ticks, sand flies) to avoid reliance on non-specific interventions, such as personal protection and landscape treatment. |
| 1. Improve understanding of vector population and pathogen heterogeneity and how this impacts control strategies. |
| 1. Improve precision of GIS, remote sensing and other technologies to establish range maps to allow for better accounting of how and when spatial and temporal effects of climate and environmental change affect vector populations and disease risk. |
