# Supplementary material for: Reframing Critical Needs in Vector Biology and Management of Vector-Borne Disease
Source: PLoS Negl Trop Dis. 2010 Feb 23;4(2):e566. doi: 10.1371/journal.pntd.0000566 (PMC2826393; doi:10.1371/journal.pntd.0000566)
Supplement: Table S3 — Resources and models. (0.10 MB DOC) [file pntd.0000566.s003.doc]

| **A. Human Resources** |
| --- |
| 1. Increase training of Master’s level personnel (e.g., MPH, MS in Entomology, MBA in Vector-borne Disease Management) for improved operational support. |
| 1. Provide in-country support for career personnel that extends beyond specific, short-term control interventions. |
| 1. Develop field sites for training personnel from both endemic and non-endemic countries in the implementation and monitoring of control programs. |
| 1. Improve interdisciplinary training for future academic mentors to encompass the disciplines of Entomology, Microbiology, Molecular Biology, Genetics, Epidemiology and Global Public Health. |
| **B. Programmatic/Research Resources** |
| 1. Increase the number of centers that focus on vector-borne disease research and training with an emphasis on reducing duplication of effort in endemic countries, improving integration of clinical studies and interventions with vector control, and providing connectivity with social scientists, economists, and environmental scientists to improve success and sustainability of control programs. |
| 1. Increase technological support centers to improve access to key expensive reagents, resources, technologies/instrumentation or assays (e.g., maintenance of transgenic organisms, synthesis of RNAi, genomic and proteomic analysis capabilities) and for assays that must be performed under biosafety containment. Epidemiologically and medically relevant materials should include transmission cycles with access to genetically diverse vectors and genetically diverse parasites. |
| 1. Enhance linkages to governmental (e.g., Department of Defense, Centers for Disease Control and Prevention, Ministries of Health) and to non-governmental international resources, facilities and infrastructure in vector-borne disease research. |
| 1. Increase support for existing reagent resource facilities (e.g., NIAID’s MR4) and improve reagent collections (e.g., cDNA clones of RNA viruses) that are relevant to vector-borne disease research and training. |
| **C. Models** |
| 1. Develop and improve training in computational, mathematical and statistical modeling for basic research, scientific inference, decision support, adaptive management, optimal allocation of limited resources, prioritizing research, experimental design for control trials, design of surveillance, and strategic planning. |
| 1. Increase collaboration among modelers and other researchers to enhance parameter estimation and access to surveillance data for use in developing and validating models. |
