# Supplementary material for: Reframing Critical Needs in Vector Biology and Management of Vector-Borne Disease
Source: PLoS Negl Trop Dis. 2010 Feb 23;4(2):e566. doi: 10.1371/journal.pntd.0000566 (PMC2826393; doi:10.1371/journal.pntd.0000566)
Supplement: Table S4 — Basic science of relevance to vector-borne diseases. (0.11 MB DOC) [file pntd.0000566.s004.doc]

| **A. Vaccines** |
| --- |
| 1. Encourage development of vaccines that can be used as part of an integrated approach for vector-borne disease control. |
| 1. Assess the impact of currently available vaccines – positive and negative – on pathogen transmission. |
| **B. Immunology and Pathology** |
| 1. Increase the study of human co-infections and co-morbidities on the response, disease progression, and transmission of vector-borne disease agents. |
| 1. Accelerate studies of the regulatory pathways that control anti-pathogen responses in insect vectors. |
| 1. Incorporate analyses of the linkages among anti-pathogen responses and other vector physiologies (e.g., lifespan, reproduction, feeding behavior) to assess the likely success of control programs that target specific vector physiologies. |
| **C. Genomics/Post-genomics** |
| 1. Increase translational studies of gene expression/proteomics for vector genomes that have been sequenced. |
| 1. For genomes that have not been sequenced, utilize new technology (e.g., 454 Sequencing™) for more rapid and less costly sequencing. |
| 1. Improve the application of new genomic and bioinformatics technologies for species and strain comparisons and proteomics/metabolomics technologies and systems biology approaches for post-genomic analyses of vectors and pathogens. |
| **D. Basic Research** |
| 1. Maintain critical basic vector biology research (e.g., identification of breeding sites, indoor resting behavior of vectors, off-host activities of vectors, ecology of immature stages) to ensure strength of pipeline from sub-cellular to organismal work and to support and improve existing control programs. |
| 1. Identify population variants with neutral markers as well as across-genome markers. |
| 1. Characterize physiological and genetic factors that regulate vector lifespan and senescence, tolerance to extreme or changing environmental conditions and habitats and that are associated with physiological adaptation to direct and indirect effects of chemical and biological controls. |
| 1. Improve SNP discovery and analytical tools for genotyping, database development, insecticide resistance detection, identification of the genetic bases for physiologies of interest and for species identification. |
| 1. For transgenesis strategies, identify more efficient transposable elements and high-throughput methods for screening of transformants and for the generation of stable targeted gene-knockouts. |
| 1. Adapt and develop analytical/bioinformatics tools to manage genomic, proteomic and other molecular databases that are accessible to the community. |
| **E. Field Research Resources** |
| 1. Improve technologies for marking insects for release-recapture studies for improved precision in determination of range, age structure, distributions, and behavior of vector populations. |
| 1. Develop non-invasive methods for age-estimation of individual vectors and estimation of age-structure of vector populations. |
| 1. Where appropriate, use semi-field systems to facilitate the transition of laboratory findings to the field. |
| 1. Support studies of arthropods of public health importance that may or may not transmit pathogens to humans (e.g., bed bugs, domestic flies, coackroaches) but that impact the sustainability and acceptability of vector control programs. |
